# Improper weapons are a neglected category of harmful objects

**DOI:** 10.1038/s41598-022-24613-8

**Published:** 2022-11-22

**Authors:** Paolo Frugarello, Elena Rusconi, Remo Job

**Affiliations:** 1 Department of Psychology and Cognitive Science, University of Trento, Corso Bettini 81, 38068 Rovereto, Trento Italy; 2 Centre for Security and Crime Sciences, University of Trento-University of Verona, Trento, Italy

**Keywords:** Psychology, Human behaviour

## Abstract

According to legislation, objects are typically classified as weapons if they are offensive per se (referred to here as proper) and if they are adapted for use as weapons or carried with the intent of causing injury (referred to here as improper), with specific regulations on their usage and possession in public spaces. However, little evidence exists on the validity of this distinction in psychology, despite a widespread recognition of the importance of psychological states and subjective perceptions in risk assessment. We conducted an online survey to evaluate hazard perceptions in relation to three dimensions (dangerousness, frequency of events, controllability) of three object categories: proper weapons, improper weapons, and everyday objects. The data from our 300 respondents reveal that the three categories of objects differ from one another on the three dimensions. Moreover, hazard perceptions differ between males and females for improper weapons but less so for proper weapons. These findings suggest that proper and improper weapons are two psychologically distinct categories, albeit with fuzzy boundaries. Investigations into their differential properties may thus help improve risk assessment in security contexts.

## Introduction

A physical offence committed against an individual is punishable according to the rules set forth in the penal code of the State in which the crime occurs, with a penalty equal to the severity of the crime committed^1,2^. In almost half the cases, physical offences are committed by using one or more harmful objects. Guns are used in four out of ten killings worldwide, blunt objects in a third, and sharp objects in a quarter^3^. In Italy, 45% of homicides in 2012 featured the use of firearms or handguns^4^. The Italian Penal System, in line with international standards (e.g. for the United Kingdom, see^5,6^; for the United States, see ^7–9^; for the District of Columbia, see^10^) distinguishes between objects that are specifically designed to offend^1^ and objects that, while not originally meant for this purpose, have still the potential to injure people^1^ (i.e. improper weapons, e.g. hammers, knives; art. 4 of Italian law n.110, April 18, 1975). The distinction is significant because the type of weapon used might hold clues as to whether the offence was perpetrated in a purposeful, premeditated or accidental way^11–13^ (see also, People v. Holt, 153 P.2d 21, 34 (Cal. 1944); People v. Smith, 104P.2d 510 (Cal.1940)), with firearms being associated with premeditation^11^ and knives with impulsivity^14^. Both proper and improper weapons are conceived as potential bearers of awful outcomes (hazard) with a variable chance of occurring (probability), based on their physical properties, such as the presence of a sharp or blunt surface^15^, and may be associated to variable levels of risk, defined as the product of severity of consequences and probability^19^. Their use and possession are thus restricted in public places or transport. For example, they are not allowed in carry-on bags in the cabin of airplanes^16–18^, as they may pose a security threat due to the accessibility of hand luggage in flight.

Despite the widely recognized relevance of psychological states and perceptions in security, safety and forensics^19,20^, research on how people perceive hazardousness in relation to objects is scarce. Of the few existing studies within the domain of weapon perception, only one^21^ focuses on the perceived hazardousness of firearms, by investigating if they are viewed as affording safety or danger, while all the others focus on the protective capacity of firearms^22,23^ or what motivates individuals to own them^24–26^. Further, no research exists on hazard perception for improper weaponry.

On the other hand, plenty of studies have investigated perception of danger and risk for a variety of contexts including natural events such as floods^27^, volcanoes^28^, diseases^29^, and technology, such as biotechnology^30^, cyberspace^31^, information security^32^, nuclear energy^33,34^, chemicals^35^ and radiation^36^. In these studies, perceptions about relevant events are investigated through a series of queries on—among others—the perceived dangerousness of the event, the degree of concern associated with each risky event, individual knowledge, feelings of indignation, social trust in public authorities, attitudes and beliefs. The individual ratings are then analyzed to identify which factors contribute to the cognitive aspect of risk perception (risk assessment).

In this study we used a series of queries to measure hazard perception in connection with three classes of objects: proper weapons, improper weapons and everyday objects. To have a subjective assessment of potentially hazardous events is critical for two reasons: research indicates a discrepancy between subjective perception and objective risk^37^; security systems benefit from taking into account subjective risk perception in hazard assessment^38–40^. Popular models for calculating objective risk are often approximations of what constitutes a given hazard and do not take into account a comprehensive list of subjective components^37^. However, both individual and social factors have been proven to influence risk perception (see e.g. the psychometric paradigm^37,38,41^). Demographic variables (e.g. gender^42–44^ and prior experiences^38^), psychological and behavioral aspects^33^, such as perceived dangerousness of an event^38^, perceived frequency of its occurrence^45,46^, and perceived controllability of the scenario^47,48^, all influence subjective risk perception^49–52^ (but see^20,49^ where the effect of demographic variables, including gender, in the perception of risk appears reduced). Furthermore, psychological and behavioral factors interact, so that an event is seen as more dangerous if it occurs more frequently or is perceived as less controllable^37,51,53–55^.

When confronted with a threat, that is a specific object or a situation that corresponds to a rising level of danger within a given context, people's behavior and reactions are based on their perceptions rather than on objective evaluations^43^. If perception is faulty, risk overestimation can lead to the unnecessary avoidance of a related behavior, while risk underestimation can wrongly encourage people to take unsafe behaviors^43,49,56^. Therefore, the more one knows about the mechanisms underlying a particular hazard, the more predictable and accurate his/her risk perceptions tend to be. This has important implications in risk communication, since a better understanding of how to increase relevant knowledge can help people make more informed and better calibrated judgments about the various risks they face^56,57^. In addition, a better understanding of individual risk perception leads to improvements in security systems, by fine-tuning risk management models so that they can more accurately predict people's behavior^40^. Furthermore, an incorrect or incomplete estimate of risk perception can strongly affect downstream processes such as risk assessment and the implementation of security models^58^.

Knowledge on the perception of weapon hazard is currently scarce and, in particular, there is a shortage of data on the distinction between proper and improper weapons. However, these may represent conceptually and practically distinct categories for several reasons. Indeed, proper and improper weapons may differ quite widely with respect to their status and availability among people; improper weapons are owned widely without any official documentation or license (art. 4 of Italian law n.110, April 18, 1975) and they are used more and more frequently as instruments for terror attacks on bystanders by lone offenders. Of the ten jihadist attacks that took place in Europe in 2020, only one involved firearms. In six of the ten attacks, a knife or another blade improper weapon was used. In the remaining three attacks, arson (1) and vehicles (2) were used^59^.

Here we have focused in particular on proper and improper weapons that could fit into hand or hold luggage, in order to maintain a consistent range of size^60^ and to use reasonably common items with clear guidance on the hazard they entail (see “Methods”). We have conducted an online survey to investigate whether specific psychological elements of the risk assessment process, such as the perceived degree of danger (hereafter, dangerousness), frequency of involvement in harmful events (hereafter, frequency of events), and feeling of control (hereafter, controllability) differ between proper weapons (e.g. firearm, bomb), improper weapons (e.g. knife, hammer) and neutral control objects (hereafter, everyday objects), such as garments or everyday tools (e.g. cap, bottle). The approach is comparable to that adopted by studies exploring the perception of objects through individual judgments on a series of attributes^61^. We also investigated whether gender has an impact on how these three categories are perceived in terms of dangerousness, frequency of events, and controllability. Despite the fact that research reveals that women are more risk averse than men in the sphere of physical health and safety^62–66^, the role of gender in subjective risk perception remains unclear and no evidence exists on the impact of gender even on the perception of hazard with proper weapons as stimuli. We will check whether the different psychological facets of the risk assessment process under examination (i.e. dangerousness, frequency of events, and controllability) correlate with one another for proper and improper weapons. As evidenced by the literature on subjective risk perception, hazard perception for an event is heightened by a perception of higher frequency of occurrence and of lower control^38,51,53,54^, but there is no data on the existence of similar relationships for proper and improper weapons. Our specific hypotheses can be summarized as follows:

H1: Object categories (proper weapons, improper weapons, everyday objects) differ in subjective judgements of dangerousness, frequency of events, controllability both between- and within-category. Proper weapons are perceived as significantly more dangerous, more frequently involved in harmful events, less controllable than improper weapons which, in turn, differ from everyday objects for a higher degree of perceived dangerousness, higher frequency of events, lower controllability.

H2: Gender has a significant impact on subjective judgements of dangerousness, frequency of events, and controllability for proper and improper weapons. Women regard proper and improper weapons as more dangerous, more frequently involved in harmful events, and less controllable than men do.

H3: In both the proper and the improper weapon categories, perceived dangerousness is positively correlated with the perceived frequency of events, and negatively correlated with the perceived level of controllability.

## Results

Data were collected from 300 individuals in an online survey, with the main focus being on the investigation of danger perception in proper weapons, improper weapons, everyday objects (henceforth, Category). The questionnaire was divided in two blocks: the first block consisted of an evaluation task in which participants used a seven-point Likert scale to rate the degree of dangerousness, frequency of events (i.e. the frequency with which these items are involved in harmful events), and controllability (henceforth, Judgment) connected with each object; the second block consisted of a written naming (identification) task. Within each block, whose order was fixed across participants, items were presented randomly. For each participant, only ratings of correctly identified items were included in the analysis. A 2 × 3 × 3 mixed ANOVA was conducted to assess whether the judgements differed across the three categories and/or gender. Pearson's correlation was then utilized to evaluate the relationship between perceived dangerousness, frequency of events, and controllability for each category. Finally, two cluster analyses were performed to explore differences and similarities within the improper weapon category and between the proper and improper weapon categories.

### ANOVA

Only 0.12% of the total observations (i.e. 48 out of 40,500 judgments, linked to 16 items across 13 participants overall) had to be excluded from the analyses due to incorrect identification. No significant deviations from normality were found (all *ps* > 0.05) but sphericity could not be assumed (all *ps* < 0.05). Therefore, results are reported after Greenhouse–Geisser correction. The mixed-design ANOVA revealed a main effect of Judgment [*F*(1.52, 453.02) = 103.79, *p* < 0.001, η_p_^2^ = 0.26] and Category [*F*(1.86, 553.27) = 630.54, *p* < 0.001, η_p_^2^ = 0.68] but not of Gender [*F*(1, 298) = 0.58, *p* = 0.45, η_p_^2^ = 0.002]. The main effects were qualified by significant two-way interactions between Gender and Judgement [*F*(1.52, 453.02) = 28.29, *p* < 0.001, η_p_^2^ = 0.09], Gender and Category [*F*(1.86, 553.27) = 4.77, *p* = 0.01, η_p_^2^ = 0.01], Category and Judgement [*F*(2.80, 834.64) = 4154.89, *p* < 0.001, η_p_^2^ = 0.93]. A significant three-way interaction between Gender, Category and Judgment [*F*(2.80, 834.64) = 15.80, *p* < 0.001, η_p_^2^ = 0.050] was also found. A post-hoc power sensitivity analysis was performed using the software program G*Power 3.1^67^. The power analysis indicated that with 300 participants, an effect size of f = 0.23 with η_p_^2^ = 0.05 and with an alpha = 0.02, we had power (equal to 1) for the three-way interaction between Gender, Category and Judgement. Bonferroni’s post-hoc tests for the two-way interaction between Judgment and Category (mean differences, pairwise comparisons test statistic, and effect size are reported in Table 1) revealed that proper weapons were significantly perceived as more dangerous than both improper weapons (p < 0.001) and everyday objects (*p* < 0.001), and that improper weapons were perceived more dangerous than everyday objects (*p* < 0.001). Ratings for frequency of events were higher for proper weapons than improper weapons (*p* < 0.001) and everyday objects (*p* < 0.001), and were higher for improper weapons than everyday objects (*p* < 0.001). In addition, pairwise comparisons revealed that the perceived controllability for proper weapons was significantly lesser than for improper weapons (*p* < 0.001) and everyday objects (*p* < 0.001), and was lesser for improper weapons than everyday objects (*p* < 0.001).

**Table 1:** Pairwise comparisons and related test statistics are reported.

Pairwise comparisons	Mean difference	Test statistics (t)	Adjusted p value	Cohen’s *d*
Proper weapons_dangerousness vs Improper weapons_dangerousness	**0.60**	**t (299) = 13.82**	***p* < 0.001**	***d* = .72**
Improper weapons_dangerousness vs Everyday objects_dangerousness	**3.08**	**t (299) = 55.44**	***p* < 0.001**	***d* > 2**
Proper weapons_dangerousness vs Everyday objects_dangerousness	**3.68**	**t (299) = 99**	***p* < 0.001**	***d* > 2**
Proper weapons_frequency of events vs Improper weapons_frequency of events	**0.51**	**t (299) = 12.35**	***p* < 0.001**	***d* = .56**
Improper weapons_frequency of events vs Everyday objects_frequency of events	**2.25**	**t (299) = 44.26**	***p* < 0.001**	***d* > 2**
Proper weapons_frequency of events vs Everyday objects_frequency of events	**2.77**	**t (299) = 49.31**	***p* < 0.001**	***d* > 2**
Proper weapons_controllability vs Improper weapons_controllability	**− 0.74**	**t (299) = − 17.27**	***p* < 0.001**	***d* = 0.86**
Improper weapons_controllability vs Everyday objects_controllability	**− 3.10**	**t (299) = − 54.29**	***p* < 0.001**	***d* > 2**
Proper weapons_controllability vs Everyday objects_controllability	**− 3.84**	**t (299) = − 86.5 **	***p* < 0.001**	***d* > 2**

Effects that remained significant after Bonferroni post-hoc tests are highlighted in bold.

**Figure 1:**
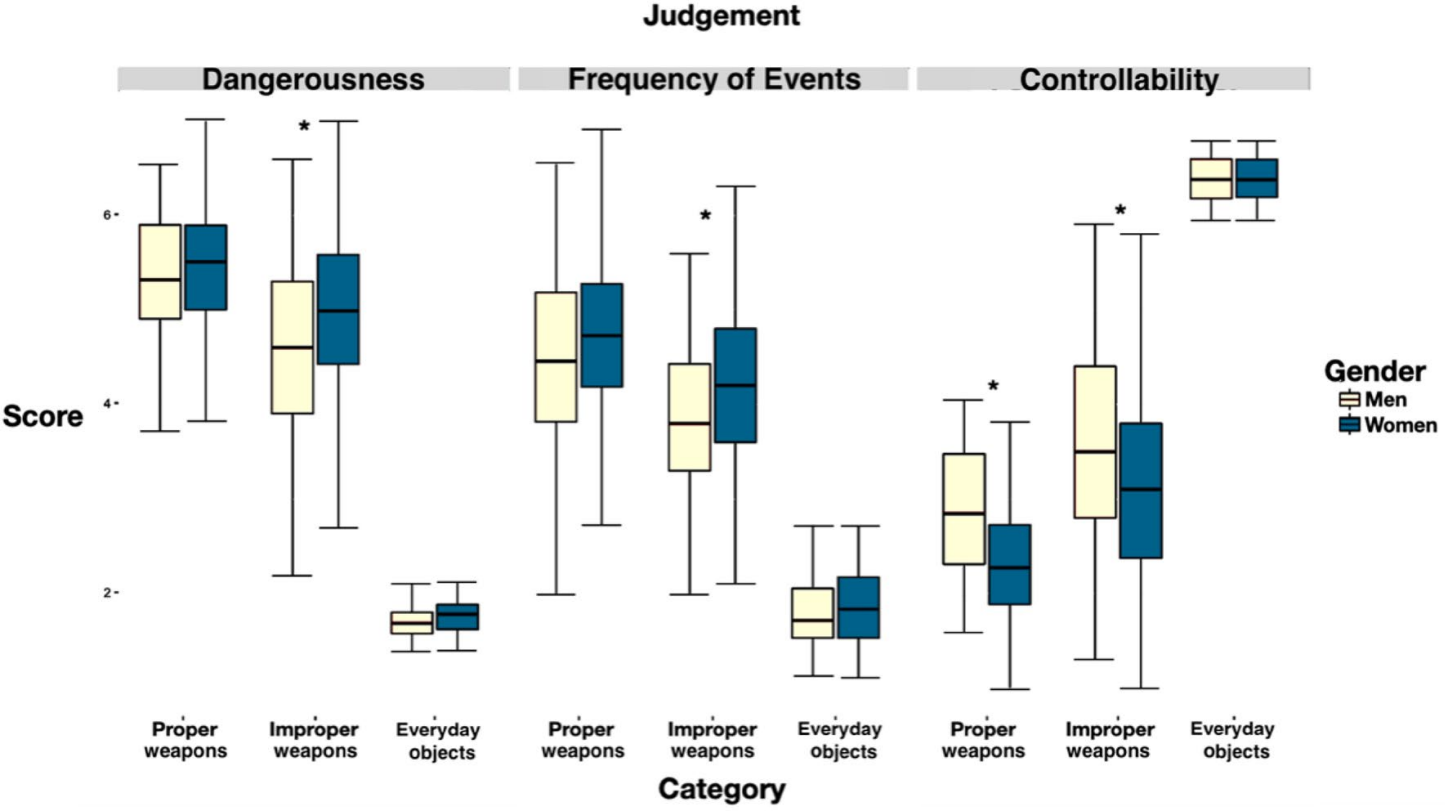
For the three categories, the effect of gender on subjective perceptions of dangerousness, frequency of events, and controllability is shown. The significance (*p* < .001) of the gender comparison of each subjective perception for a specific category is indicated by asterisks.

Bonferroni’s post-hoc tests for the three-way interaction revealed that a Gender effect was consistently present with improper weapons, as shown in Fig. 1. Means, standard deviations and test statistics are reported in Table 2. Compared to women, men judged improper weapons less dangerous (*p* < 0.001), less frequently involved in harmful events (*p* < 0.001), and more controllable (*p* < 0.001). For proper weapons, men had a higher sense of control over proper weapons (*p* < 0.001) but they did not differ from women in the perceived dangerousness (*p* = 1) and the perceived frequency of events (*p* = 0.22). For everyday objects, no gender difference emerged in any of the three dimensions of dangerousness, frequency, and controllability.

**Table 2:** Means and standard deviations, of each pairwise comparison for Gender.

Pairwise comparisons	Male *M (SD)*	Female *M (SD)*	Test statistics (t)	Adjusted p value	Cohen’s *d*
Proper weapons: Dangerousness	5.34 (0.64)	5.45 (0.63)	t (253.07) = − 1.44	*p* = 1	*d* = 0.17
Proper weapons: Frequency of events	4.44 (0.99)	4.69 (0.85)	t (227.88) = − 2.27	*p* = 0.22	*d* = 0.27
**Proper weapons:** **Controllability**	**2.87 (0.68)**	**2.34 (0.69)**	**t (261.92) = 6.59**	***p* < 0.001**	***d* = 0.77**
**Improper weapons:** **Dangerousness**	**4.54 (1)**	**4.99 (0.92)**	**t (240.43) = − 3.87**	***p* < 0.001**	***d* = 0.46**
**Improper weapons:** **Frequency of events**	**3.82 (0.84)**	**4.24 (0.92)**	**t (270.85) = − 4.06**	***p* < 0.001**	***d* = 0.47**
**Improper weapons:** **Controllability**	**3.61 (0.92)**	**3.08 (0.93)**	**t (256.85) = 4.87**	***p* < 0.001**	***d* = 0.57**
Everyday objects: Dangerousness	1.70 (0.16)	1.75 (0.18)	t (273) = − 2.69	*p* = 0.07	*d* = 0.31
Everyday objects: Frequency of events	1.80 (0.39)	1.84 (0.42)	t (265.98) = − 0.83	*p* = 1	*d* = 0.10
Everyday objects: Controllability	6.40 (0.25)	6.39 (0.24)	t (250.03) = 0.25	*p* = 1	*d* = 0.03

In addition, test statistic, adjusted p value and Cohen’s *d* are reported. Effects that remained significant after Bonferroni post-hoc tests are highlighted in bold.

### Correlations

The relationship among the dimensions of interest was then examined using Pearson's correlation coefficients. A Bonferroni correction was applied to the overall alpha threshold, which was thus lowered to 0.0022 for a single test. Results showed that proper weapons’ dangerousness correlates positively with frequency of events, r(298) = 0.58, *p* < 0.001, and negatively with controllability, r(298) = − 0.42, *p* < 0.001. The same pattern was found for improper weapons, in which dangerousness correlates positively with frequency of events, r(298) = 0.62, *p* < 0.001, and negatively with controllability, r(298) = − 0.41, *p* < 0.001. The subjective perception of the frequency of events is negatively correlated with controllability both for proper, r(298) = − 0.29, *p* < 0.001, and improper weapons, r(298) = − 0.34, *p* < 0.001. Post-hoc power sensitivity analyses for each of the above correlation were performed using the software program G*Power 3.1^67^. The power analysis indicated that with 300 participants, correlation coefficients of r_proper_dangerousness&frequency_of_events_ = 0.58, r_proper_dangerousness&controllability_ = 0.42, r_proper_frequency_of_events&controllability_ = 0.29, r_improper_dangerousness&frequency_of_events_ = 0.62, r_improper_dangerousness&controllability_ = 0.41, r_improper_frequency_of_events&controllability_ = 0.34, an alpha = 0.0022, we had power (1) for the correlation analyses, except for the correlation between frequency of events and controllability in proper weapons (power of = 0.99).

### Cluster analyses

Given the significant difference between proper and improper weapons for dangerousness, frequency of events, and controllability, two cluster analyses were performed to better characterize the categories under investigation^68^. Cluster analyses were carried out using R (4.0.3), with the “Cluster” (2.1.0), “Factoextra” (1.0.7) packages. A first cluster analysis aimed to identify any subsets within the improper weapons category. A second cluster analysis was performed to identify possible connections/overlaps between the two main categories of interest (proper weapons and improper weapons). Both analyses were based on dangerousness, frequency of events and controllability judgments, which served as input.

For each cluster analysis, we first conducted agglomerative hierarchical cluster analyses using Ward's method of minimum variance with a squared Euclidean distance measure^69^. The best-distinguished cluster solution was then determined from the visual inspection of the resulting dendrogram, a tree diagram showing the arrangement of the clusters produced by hierarchical clustering. After identifying the number of distinct clusters, k-means clustering with the number of clusters as input for k was used to determine the content of the distinct groups.

The analysis for the improper weapons category yielded a four-cluster solution, with hammer and knife in cluster A, cutter, razor, saw, scissors, blowpipe, and firecrackers in cluster B, screwdriver, cooking thermometer, tube, baseball bat and wrench in cluster C, pliers and chisel in cluster D. The levels of dangerousness and frequency of events decrease as the degree of controllability increases, from cluster A to cluster D.

**Figure 2:**
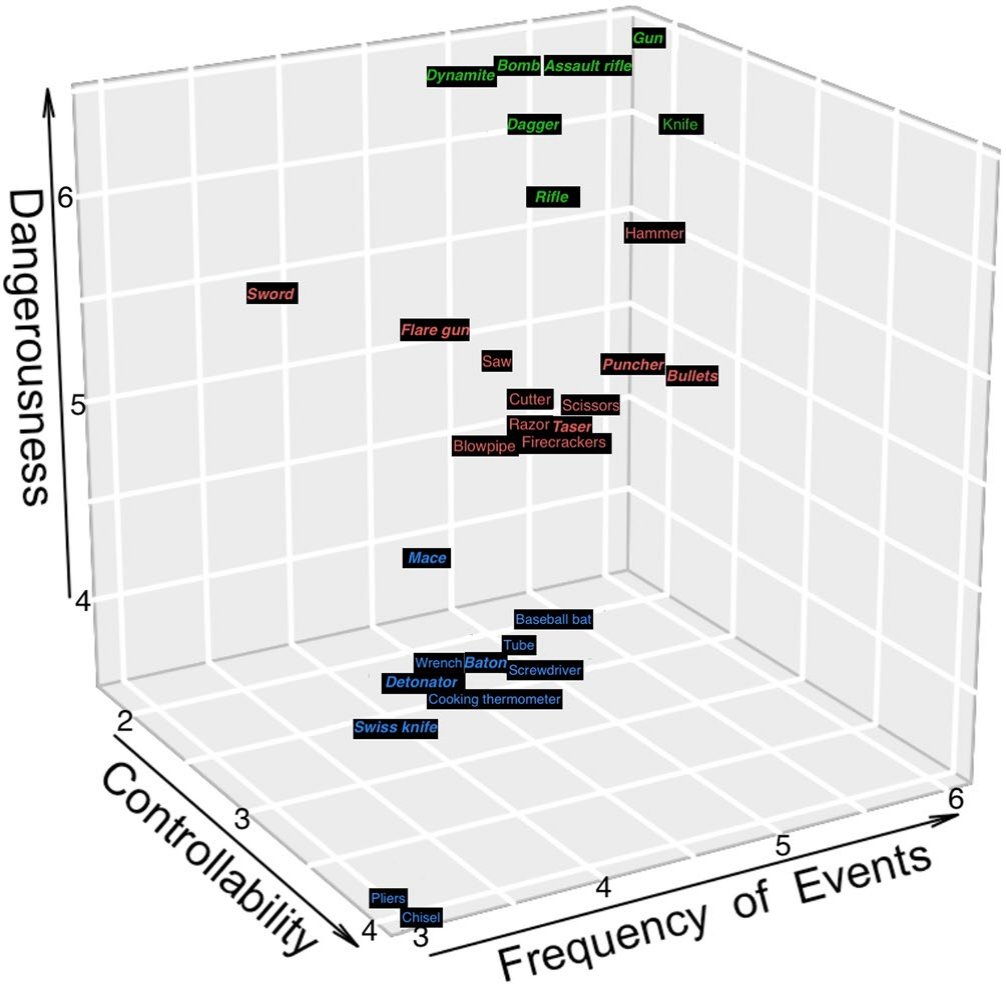
The three clusters of proper and improper weaponry are distributed in three dimensions for dangerousness (y-axis), frequency of events (x-axis), and controllability (z-axis). A different colour denotes affiliation with each of the clusters: Cluster A′ is green, cluster B′ is red, cluster C′ is blue. To distinguish proper from improper weapons, the names of proper weapons are italicized and bolded.

The analysis with both the proper weapons and the improper weapons categories yielded a three-cluster solution, with a peak of overlap between categories in the middle cluster, as shown in Fig. 2. Cluster A′ includes six proper weapons (gun, assault rifle, rifle, bomb, dynamite, dagger) and two improper weapons (hammer, knife); cluster B′ includes five proper weapons (sword, flare gun, puncher, bullets, taser) and six improper weapons (saw, cutter, scissors, razor, blowpipe, firecrackers); cluster C′ contains four proper weapons (mace, baton, detonator, Swiss knife) and eight improper weapons (baseball bat, tube, screwdriver, wrench, cooking thermometer, chisel and pliers).

Again, the levels of dangerousness and frequency of events decrease as the degree of controllability increases, from cluster A′ to cluster C′.

## Discussion

Research on risk perception focuses on several areas (e.g. risk exposure, risk communication etc.). In this study we investigated the subjective feeling of whether different kinds of objects, namely proper weapons, potentially dangerous utensils (improper weapons), and non-dangerous everyday objects, may be a potential cause of harms. Studies on proper weapons are scarce and there appear to be even fewer studies on improper weaponry, which encompass work equipment or utensils with the potential to cause harm, despite the fact that they are not designed for it. To address this gap, we devised an online survey in which we collected judgments on dangerousness, as well as controllability and frequency of events, of improper weapons, proper weapons, and everyday objects.

The main finding of the study is that people's perceptions of hazard, of the frequency of criminal incidents involving them, and of their sense of controllability differ widely across proper weapons, improper weapons, and everyday objects. On all three dimensions, improper weapons differ both from proper weapons and from everyday objects: on the one hand, they are perceived as significantly less deadly, less frequently involved in harmful events and more controllable than proper weaponry; on the other hand, they are perceived as significantly deadlier, more frequently involved in harmful events, and less controllable than everyday objects.

The difference we found supports the validity of the categorical distinction between proper and improper weapons in the Italian and international legislation, and attests to the need for an analogous distinction between these two categories in the risk assessment literature.

The observed disparity can be explained in light of studies on the subject of risk perception^38,46,52^, which map a wide range of risks in a two-dimensional factor space according to whether they were unknown and feared. The dimension of risks increases when the sensation of dread and the difficulty of control increase, along with a greater sensation of frequent involuntary exposure^46^. Thus, proper and improper weapons pose a higher risk than everyday objects because they are perceived as more dangerous and less controllable (that is, the severity of consequences), and more frequently involved in harmful events (that is, the probability of occurrence). Our assessment that there are two psychologically separate categories of weapons—proper and improper weapons—leads us to believe that the level of risk they pose is likewise distinct, and that people may act differently when confronted with either type of weapon, highlighting the need for additional research. Indeed, we found a positive correlation between dangerousness and frequency of events and a negative correlation between dangerousness and controllability and we hypothesise that frequency of events and controllability may have led to a higher degree of perceived dangerousness for both proper and improper weapons. In addition, we hypothesise that the lower degree of controllability of proper weapons, as well as the perception of a higher frequency of accidents involving them, may influence the perception of dangerousness of proper weapons compared to improper weapons; the same pattern can be found in the difference between improper weaponry and everyday objects.

Two cluster analyses indicated stratifications within the category of improper weapons and overlaps with the category of proper weapons. A first cluster analysis led to the identification of four clusters in the improper weapons category.

When looking at the composition of the clusters, knife and hammer appear at the opposite end of the spectrum compared to chisel and pliers. Sharp (e.g. the knife) and blunt things (e.g. the hammer) are the most commonly used improper weapons in criminal episodes (for Italy see^70^; for the U.K. see^71^; for South Korea see^72^), while the chisel and pliers are much less used in harmful events^15^. The overall higher dangerousness of the knife and hammer might also be due to associated perceptions of higher severity of injuries caused by these objects^15,73^. The remaining objects are divided into two intermediate clusters and exhibit a wide range of physical characteristics (elongated, sharp, blunt shape, heavy or light).

Notably, the overall level of perceived dangerousness appears to decrease as an object's use becomes more specialized. Therefore, objects with more diverse uses (e.g., the knife, the hammer) are considered more dangerous than objects with more sectoral and specialized uses (e.g. the chisel). The second cluster analysis shows that the categories of proper and improper weapons have a graded structure just like natural categories do^74^. Objects in a category lie on a gradient of typicality, ranging from extremely good to very poor examples of the category^75^, with prototypical members serving as reference point to which other category members can be assessed^76^. Thus, categories vary for the degree of membership of their exemplars and they may even overlap other categories, as is the case with the categories of proper and improper weapons under examination.

As predicted, we found that gender influence risk perception of weapons; however, the pattern we found is rather articulated, suggesting that it may not be gender per se that drives the effect but rather everyday experience with the type of objects to be judged. To summarize, women judge improper weapons, but not proper weapons, riskier and more involved in events than men do, and they judge both improper and proper weapons less controllable than men do.

We suspect that the greater use of work tools by males than by women^77^ may explain the gender difference found in the perception of risk in improper weapons and the controllability of proper weapons. In Italy at least, this may be indirectly linked to gender disparity in employment^78^. Among the workforce in the private sector where a wide unbalance between men and women is found^79^ (Decree-Law n.142, October 16, 2020) figure craft workers, engineers, and installation and maintenance experts of electrical and electronic equipment for 2021 (95.1% male–female disparity). Intriguingly, in Brodeur et al.^61^, tools (including those that we classify as improper weapons) were the only inanimate stimuli showing a significant gender difference, males being more familiar with tools than females. This in turn may reflect gender differences in interests and exposure to experiences ingrained in Western culture that may be based only partly on biology^8^. Males' larger use and familiarity with improper weapons may influence them to associate more strongly these objects to their original function, a sort of functional fixedness effect^80^, leading men to perceive them as less threatening than women do. Individuals' familiarity with the way objects are typically used, according to Duncker^80^, hinders people from representing them under a new aspect or purpose. In this case, previous experiences that stress the conventional function^81,82^ of hand tools might minimize males' connection of these objects with their alternative function as weapons.

Men and women differ widely also with respect to owning a proper weapon (for the United States, see^83^) which may lead to the divarication of the sense of control about them between women and men. No data exist for civilian weapon ownership for Italy, but the imbalance between men and women in the armed forces^84^, and in hunting practices, and thus the differential in the authorized holding of proper weaponry, can serve as an indirect validation. Because proper weapons have a single function, the phenomenon of functional fixedness would be missing in this scenario, and the gender difference in the controllability could originate from women's poorer familiarity with weapons compared to males, as there are fewer women in possession of a weapon than men^83^.

By testing larger and more heterogeneous samples of participants it would be possible to ascertain the general character of the psychological distinction between proper and improper weaponry we have documented here. Additionally, the evaluation of extra features could help enrich our model and ponder on the wider implications of such distinction. Indeed, the disparities in risk perception of proper and improper weaponry that we report may have substantial implications for risk communication, in addition to their importance for risk assessment. Risk communication aims to bridge the knowledge gap between experts and non-experts, as naive people frequently lack proper awareness of the dangers they confront^46^ and perceive potential hazards more erroneously than experts^33,37,38,41^. The increment of the relevant knowledge among people would result in a more informed and better calibrated judgments about the existence, nature, and/or severity of risks and hazards and more predictable and highly correlated (with domain-specific knowledge) risk perceptions^46,85^.

## Methods

### Stimuli collection and standardization

For the Italian legal system^1^ (see also art. 1,2,4 of Italian law n. 110, April 18, 1975), the following are considered proper weapons: any sort of firearm (including ammunition); air-powered weapons; sidearms (such as swords, double-edged knives or switchblades, daggers, etc.); instruments for which there is an absolute prohibition (maces, punchers; batons are considered as proper weapons by decision no. 22314, July 8, 2019, of the Italian Supreme Court); bacteriological or chemical weapons (not included in this survey); all incendiary, exploding, or disrupting devices (bombs, Molotov cocktails). According to the same Italian legislation, improper weapons include blunt objects like clubs, tubes, chains, bolts, metal balls, hammers, and bars; cutting tools like kitchen knives or hatchets, are additional examples of improper weapons. Arches, blowguns and crossbows are also considered as improper weapons: they are recreational and hunting tools, hence should only be used for such purposes. For each of the classes of objects (apart for bacteriological and chemical weapons), at least two examples were chosen and included as items in the current study (i.e. two firearms, two cutting weapons, etc.).

For the purpose of this study, the distinction between everyday objects and weapons is based on the regulations of the Italian Civil Aviation Authority (available here: https://www.enac.gov.it/passeggeri/cosa-portare-bordo/articoli-vietati-in-cabina). Everyday objects were chosen among those allowed in a plane's cabin, while items from the other two categories are not allowed. Based on this distinction, clothing, accessories, toys, reading material (i.e. books, magazines), and plastic containers (i.e. glasses, bottles, plastic bags) are examples of everyday objects. Also for each of these subgroups at least two specimens were used.

Overall, 15 items for each of the three categories were selected.

Item selection was determined by physical dimensions and frequency of use in the Italian language, so that all the objects selected have comparable physical dimensions that allow them to be carried in a hand luggage and relatively high frequency of use in the Italian language, avoiding regional, dialectal, obsolete and low-use terms (as found in the Nuovo Vocabolario di base della lingua italiana, NVdB, New Basic Vocabulary of the Italian Language, available here: dizionario.internazionale.it/nuovovocabolariodibase; see Supplementary Table S1 for details). As a result, the following items were included in our study:

*Proper weapons*: mace, flare gun, bomb, dagger, puncher, taser, dynamite, bullets, baton, detonator, gun, sword, Swiss knife, rifle, assault rifle;

*Improper weapons*: baseball bat, cooking thermometer, cutter, screwdriver, hammer, tube, firecrackers, pliers, chisel, razor, scissors, knife, blowpipe, wrench, saw;

*Everyday objects*: glass, hat, jacket, ball, bottle, sock, shirt, book, cap, undershirt, bag, skirt, heel shoe, jeans, sneakers.

Photographs of these items were obtained via Google image search and selected among the files licensed with Creative Commons 1.0 Universal (CC0 1.0). Colored backgrounds or shadows, if present, were removed from the figures so that all items stood out against a white background, and a single object was shown per image. The images were resized (maintaining original proportions) and their dimension was standardized to 350 pixels (wide) × 500 pixels (high). As dangerous objects are seen as more hazardous when they are oriented towards participants than when they are directed away^86^, the orientation of proper and improper weapons was made uniform (from the participant's perspective, the weapon's handle—where present—is on the left side of the screen, and the offensive part is directed towards the right), in order to eliminate potential biases in hazard perception. Also, the orientation of everyday objects with a handle was made uniform and consistent with the criteria applied to weapons. For each image we calculated three standard visual complexity indices: edge density, feature congestion and subband entropy^87–89^. With a significance criterion of p < 0.02, a 3 × 3 repeated-measures ANOVA shows that the three categories do not differ from each other on these complexity measures. The values of the indices for each image are reported in Supplementary Table S1.

### Participants

Participants were recruited via social networks and personal contacts at the Universities of Trento, Siena and Venezia (Italy). A total of 300 Caucasian adults (all of whom were over the age of 18) Italian speaking volunteers (120 males, *M*_age_ = 32.06, *SD*_age_ = 10.84; 180 females, *M*_age_ = 30.64, *SD*_age_ = 11.48) were included in the study. Prior to the experiment, all participants provided online informed consent and they received no compensation for their participation. All methods were approved by the University of Trento, Human Research Ethics Committee (Protocol 2020-019). The whole procedure was realized in accordance with the Helsinki Declaration.

### Procedure

Participants were granted access to the Qualtrics survey platform via an open web link. First, they were provided with general information on the questionnaire. The rationale and the purpose of the study were made clear, with the main focus referred to as the investigation of danger perception in several categories of objects. It was specified that these objects could have been either weapons or everyday life objects. In addition, the following definitions of danger and weapon were given, in order to prevent misjudgments:

*Danger:* “Danger is defined as an intrinsic trait or quality of a given factor that has the potential to inflict damage, as defined by art. 2, letter r, of Italian law n. 81, April 9, 2008. Danger is thus an intrinsic attribute (of the situation, object, substance, etc.) unrelated to external circumstances; it is a scenario, object, substance capable of hurting people, due to its properties or characteristics”.

*Weapon*: “Strictly from a technical standpoint, a weapon is any instrument capable of offending, either by its natural purpose or by the method in which it is used”.

The questionnaire was divided in two blocks. The first block required evaluating the degree of dangerousness, controllability and frequency of events associated with each object; the second block consisted of a written naming task. The 45 pictures of the study items were shown once in the first block and once in the second block. The order of blocks was fixed across participants. Within each block, items were presented randomly.

**Figure 3:**
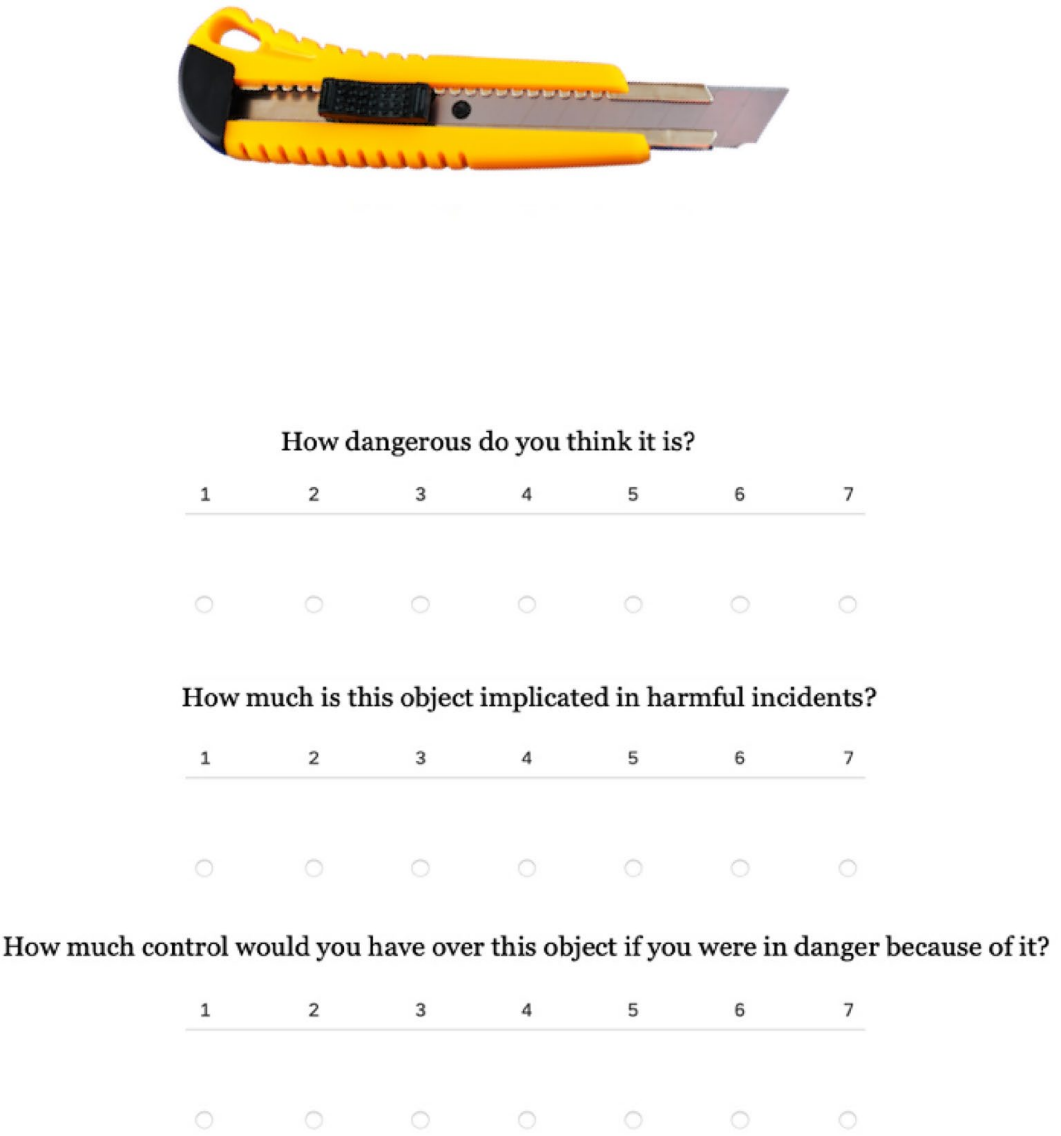
A page from the questionnaire relating to the perceived dangerousness, frequency of events, and controllability for the stimulus "cutter" is reported here. When reporting the questionnaire page in this figure, the gap between the questions was decreased, to optimize the use of space.

In the evaluative task, the picture was presented at the top center of the page under which three questions appeared (Fig. 3).

Participants were asked to judge the extent to which the objects of the kind displayed could pose a danger (dangerousness; "How dangerous do you think it is?"), how common are harmful episodes involving the object (frequency of events “How much is this object implicated in harmful incidents?”), what level of control participants would be able to exert on that object in the context of an event (controllability; "How much control would you have over this object if you were in danger because of it?”). A seven-point Likert type scale (1 = Not at all to 7 = Extremely) was provided to answer each question. Participants were required to answer all questions in order to proceed to the next page. Once moved on to the next page, participants could not go back and amend their responses.

In the written naming task, each image was displayed in the top center of the page, with a text box below it where the participant could type the name of the item shown. Participants were asked to write the name of the item, even in case of uncertainty. After providing at least one response, they could then move on to the following page. The purpose of the written naming task was to ensure that participants could actually identify what they were judging; items for which an incorrect name was provided were excluded from the analyses.

In the final section of the questionnaire, participants were asked to provide demographic information about their age, gender and education. The IP address of the client computer was used to identify potential duplicate entries from the same user.

### Statistical analysis

Statistical analyses were carried out using R (4.0.3), with the “Ez” (4-4.0), “Psychreport” (3.0.1), “Effectsize” (0.4.5), “Corrplot” (0.92) packages. The analyses were performed on assessments for dangerousness, frequency of events, controllability (henceforth, Judgment) of the three categories of objects: proper weapons, improper weapons, everyday objects (henceforth, Category). For each participant, only ratings of correctly identified items were included in the analysis.

Normality and sphericity checks were carried out on the dependent variable, for any cell of the Judgement × Category design, using the Kolmogorov–Smirnov test and Mauchly’s test, respectively.

In order to assess whether the judgements differed across the three categories and/or gender, a 2 × 3 × 3 mixed ANOVA was conducted, with Gender (Women, Men) as between-participants factor, Judgment (dangerousness, frequency of events, controllability) and Category (proper weapons, improper weapons, everyday objects) as within-participants factors. A significance criterion of p < 0.02 was used. In case of significant interactions, pairwise comparisons were conducted with Bonferroni’s post-hoc test.

For each category, Pearson’s correlation was used to assess the relation between the perceived dangerousness, frequency of events and controllability. The family-wise alpha threshold was 0.02 (lowered to 0.0022 per individual test after Bonferroni correction). Finally, for the proper and improper weapon categories, two cluster analyses were conducted for exploratory purposes. For both cluster analyses, an analysis of agglomerative hierarchical clusters using Ward's method of minimum variance with a squared Euclidean distance measure was performed first, followed by an analysis of k-means clustering.

## Supplementary Information

Supplementary Information.

## Data Availability

All materials and data are available at the OSF Repository and can be accessed at https://osf.io/ghyqm/?view_only=0da5b75c625443d5a256ed78a86f113c. For any requests, please contact paolo.frugarello@unitn.it or elena.rusconi@unitn.it.

## References

[1] Italian penal code. https://www.altalex.com/documents/codici-altalex/2014/10/30/codice-penale (2022).

[2] *La Costituzione della Repubblica Italiana.* Milano: Giuffrè Francis Lefebvre (2021).

[3] UNODC. *Global study on Homicide*. United Nations publication, Sales No. 14.IV.1, Vienna (2013).

[4] Butchart A, Mikton C, Krug E (2014). Global Status Report on Violence Prevention.

[5] Offensive Weapons Act. Uk government legislation (2019). https://assets.publishing.service.gov.uk/government/uploads/system/uploads/attachment_data/file/905406/2019_08_14_OWAct_Draft_Statutory_Guidance_.pdf.

[6] Criminal Justice Act. Uk government legislation (2003). https://www.legislation.gov.uk/ukpga/2003/44/contents.

[7] Gun Control Act. 18 U. S. C. § 101 (1968). https://www.govinfo.gov/content/pkg/STATUTE-82/pdf/STATUTE-82-Pg1213-2.pdf.

[8] Federal Switchblade Act, 15 U. S. C. § 1241 (1958). https://www.govinfo.gov/content/pkg/USCODE-2011-title15/html/USCODE-2011-title15-chap29.htm.

[9] United States Code, Title 18 (2015). https://www.law.cornell.edu/uscode/text/18. 12041401

[10] Criminal Offenses and Penalties, DC Code § 22–4501 (2019).

[11] Pelletier KR, Pizarro JM (2019). Homicides and weapons: Examining the covariates of weapon choice. Homicide Stud..

[12] Cook PJ, Tonry M, Morris N (1983). (1983). The influence of gun availability on violent crime patterns. Crime and justice: An annual review of research.

[13] Cook PJ, Nagin D (1979). Does the Weapon Matter?: An Evaluation of a Weapons-emphasis Policy in the Prosecution of Violent Offenders.

[14] Mize KD, Shackelford TK, Weekes-Shackelford VA (2011). Younger women incur excess risk of uxoricide by stabbing and other hands-on killing methods. Pers. Individ. Differ..

[15] Carr DJ, Godhania K, Mahoney PF (2019). Edged weapons awareness. Int. J. Legal Med..

[16] Hancock PA, Hart SG (2002). Defeating terrorism: What can human factors/ergonomics offer?. Ergon. Des..

[17] Harris DH (2002). How to really improve airport security. Ergon. Des..

[18] Schwaninger A (2004). Increasing efficiency in airport security screening. WIT Trans. Built Environ..

[19] Slovic, P. & Weber, E. U. Perception of risk posed by extreme events. In *Regulation of Toxic Substances and Hazardous Waste,* (2nd edition; eds. Applegate, J. S., Laitos, J. G., Gaba, J. M. & Sachs, N. M.) (2013).

[20] Finucane ML, Alhakami A, Slovic P, Johnson SM (2000). The affect heuristic in judgments of risks and benefits. J. Behav. Decis. Mak..

[21] Syropoulos S (2021). Deadly but protective: Americans’ unique perception of weapons. Peace Conflict J. Peace Psychol..

[22] Buttrick N (2020). Protective gun ownership as a coping mechanism. Perspect. Psychol. Sci..

[23] Barragan M, Sherman N, Reiter K, Tita GE (2016). “Damned if you do, damned if you don’t”: Perceptions of guns, safety, and legitimacy among detained gun offenders. Crim. Justice Behav..

[24] Leander NP (2019). Mass shootings and the salience of guns as means of compensation for thwarted goals. J. Pers. Soc. Psychol..

[25] Stroebe W, Leander NP, Kruglanski AW (2017). Is it a dangerous world out there? The motivational bases of American gun ownership. Pers. Soc. Psychol. Bull..

[26] Kruis NE, Wentling RL, Heirigs MH, Ishoy GA (2020). Assessing the impact of knowledge and location on college students’ perceptions of gun control and campus carry policies: A multisite comparison. Am. J. Crim. Justice.

[27] Raaijmakers R, Krywkow J, van der Veen A (2008). Flood risk perceptions and spatial multi-criteria analysis: An exploratory research for hazard mitigation. Nat. Hazards.

[28] Haynes K, Barclay J, Pidgeon N (2008). Whose reality counts? Factors effecting the perception of volcanic risk. J. Volcanol. Geotherm. Res..

[29] Setbon M, Raude J, Fischler C, Flahault A (2005). Risk perception of the "mad cow disease" in France: Determinants and consequences. Risk Anal..

[30] Savadori L, Savio S, Nicotra E, Rumiati R, Finucane MPS (2004). Expert and the public perception of risk from biotechnology. Risk Anal..

[31] Jackson, J., Allum, N. & Gaskell, G. Perceptions of risk in cyberspace. Cyber Trust & Crime Prevention Project (2004).

[32] Vyskoc, J. & Fibikova, L. IT Users' Perception of Information Security. Proceedings, IFIP WG9.6/11.7 conference. Security and Control of IT in Society-SCITS-II, June 15–16, 2001, Bratislava.

[33] Sjöberg L (2004). Explaining individual risk perception: The case of nuclear waste. Risk Manage..

[34] Stainer A, Stainer L (1995). Young people’s risk perception of nuclear power—a European viewpoint. Int. J. Glob. Energy Issues.

[35] Liu T, Zhang H, Li X, Zhang H (2020). Individual factors influencing risk perceptions of hazardous chemicals in China. Environ. Res..

[36] Freudenberg LS, Beyer T (2011). Subjective perception of radiation risk. J. Nucl. Med..

[37] Slovic, P. The Perception of Risk. Earthscan Publications Ltd (2001).

[38] Slovic P (1987). Perception of risk. Science.

[39] Rasmussen J (1997). Risk management in a dynamic society: A modelling problem. Saf. Sci..

[40] Glendon AI, Clarke S, McKenna E (2016). Human Safety and Risk Management.

[41] Sjöberg L (2003). Distal factors in risk perception. J. Risk. Res..

[42] Cummings CL, Berube DM, Lavelle ME (2013). Influences of individual-level characteristics on risk perceptions to various categories of environmental health and safety risks. J. Risk. Res..

[43] Chauvin B, Hermand D, Mullet E (2007). Risk perception and personality facets. Risk Anal..

[44] Gustafsod PE (1998). Gender differences in risk perception: Theoretical and methodological perspectives. Risk Anal..

[45] Trumbo CW (2016). A cognitive-affective scale for hurricane risk perception. Risk Anal..

[46] Lichtenstein S, Slovic P, Fischhoff B, Layman M, Combs B (1978). Judged frequency of lethal events. J. Exp. Psychol. Hum. Learn..

[47] Rohrmann, B. Risk perception, risk attitude, risk communication, risk management: A conceptual appraisal. In *Proceedings of the 15th International Emergency Management Society* (TIEMS) Annual Conference, Prague, Czech Republic, 17–19 June 2008.

[48] Portell M, Gil MR, Losilla MJ, Vives J (2014). Characterizing occupational risk perception: The case of biological, ergonomic and organizational hazards in Spanish healthcare workers. Span. J. Psychol..

[49] Sjoberg L (2000). Factors in risk perception. Risk Anal..

[50] Boholm A (1998). Comparative studies of risk perception: A review of twenty years of research. J. Risk. Res..

[51] Slovic P, Fischhoff B, Lichtenstein S, Haimes YY (1981). Rating the risks. Risk/Benefit Analysis in Water Resources Planning and Management.

[52] Fischhoff B, Slovic P, Lichtenstein S, Read S, Cambs B (1978). How safe is safe enough? A psychometric study of attitudes towards technological risks and benefits. Policy Sci..

[53] Strobel G (1991). Cognitive determinants in the judgment of risks in the workplace. Psychol. Praxsic.

[54] Leiter, M.P. & Cox, T. The impact of stress on safe working behavior in health care: Implications for training and task design. In *Changing Workplace* (eds. Keita, G. P., & Hurrell, J. J.) (1992).

[55] Leiter MP, Zanaletti W, Argentero P (2009). Occupational risk perception, safety training, and injury prevention: Testing a model in the Italian printing industry. J. Occup. Health. Psychol..

[56] Slovic P, Finucane ML, Peters E, MacGregor DG (2004). Risk as analysis and risk as feelings: Some thoughts about affect, reason, risk, and rationality. Risk Anal..

[57] Volz KG, Gigerenzer G (2012). Cognitive processes in decisions under risk are not the same as in decisions under uncertainty. Front. Neurosci..

[58] Tamasi G, Demichela M (2011). Risk assessment techniques for civil aviation security. Reliab. Eng. Syst. Saf..

[59] Europol. European Union Terrorism Situation and Trend Report (2021) https://www.europol.europa.eu/publications-events/main-reports/european-union-terrorism-situation-and-trend-report-2021-tesat.

[60] Filliter JH, McMullen PA, Westwood D (2005). Manipulability and living/non-living category effects on object identification. Brain Cogn..

[61] Brodeur MB, Guérard K, Bouras M (2014). Bank of standardized stimuli (BOSS) phase II: 930 new normative photos. PLoS One.

[62] Hersch J (1996). Smoking, seat belts, and other risky consumer decisions: Differences by gender and race. Manage. Decis. Econ..

[63] Barsky RB, Juster FT, Kimball MS, Shapiro MD (1997). Preference parameters and behavioral heterogeneity: An experimental approach in the health and retirement study. Q. J. Econ..

[64] Pacula RL (1997). Women and substance use: Are women less susceptible to addiction?. Am. Econ. Rev..

[65] Harrant V, Vaillant NG (2008). Are women less risk averse than men? The effect of impending death on risk-taking behavior. Evol. Hum. Behav..

[66] Harris CR, Jenkins M (2006). Gender differences in risk assessment: Why do women take fewer risks than men?. Judgm. Decis. Mak..

[67] Faul F, Erdfelder E, Lang AG, Buchner A (2007). G*Power 3: A flexible statistical power analysis program for the social, behavioral, and biomedical sciences. Behav. Res. Methods.

[68] Everitt B (2011). Cluster Analysis.

[69] Ward JH (1963). Hierarchical grouping to optimize an objective function. J. Am. Stat. Assoc..

[70] Vassalini M, Verzeletti A, De Ferrari F (2014). Sharp force injury fatalities: A retrospective study (1982–2012) in Brescia (Italy). J. Forensic Sci..

[71] Henderson JP, Morgan SE, Patel F, Tiplady ME (2005). Patterns of non-firearm homicide. J Clin. Forensic Med..

[72] Park J, Son H (2018). Weapon use in Korean homicide: Differences between homicides involving sharp and blunt instruments. J. Forensic Sci..

[73] Ambade VN, Godbole HV (2006). Comparison of wound patterns in homicide by sharp and blunt force. Forensic Sci. Int..

[74] Rosch E (1975). Cognitive representations of semantic categories. J. Exp. Psychol. Gen..

[75] Barsalou LW (1985). Ideals, central tendency, and frequency of instantiation as determinants of graded structure in categories. J. Exp. Psychol. Learn. Mem. Cogn..

[76] Mervis CB, Rosch E (1981). Categorization of natural objects. Annu. Rev. Psychol..

[77] Eurostat data (2019) https://ec.europa.eu/eurostat/statistics-explained/index.php?title=How_do_women_and_men_use_their_time_-_statistics&oldid=463738#Construction_mainly_done_by_men.2C_gardening_by_both_genders.

[78] ISTAT (2021) https://www.istat.it/it/files//2021/11/Employment-and-unemployment_202109.pdf.

[79] ISTAT (2019) https://oa.inapp.org/bitstream/handle/123456789/829/INAPP_Cardinali_Gender_policies_report_2019.pdf?sequence=1.

[80] Duncker K (1945). On problem-solving. Psychol. Monogr..

[81] Bargh JA, Ferguson MJ (2000). Beyond behaviorism: On the automaticity of higher mental processes. Psychol. Bull..

[82] Bargh JA, Chartrand TL (1999). The unbearable automaticity of being. Am. Psychol..

[83] Wolfson JA, Azrael D, Miller M (2020). Gun ownership among US women. Inj. Prev..

[84] Italian Ministry of Defence (2019). http://www.esercito.difesa.it/Rapporto-Esercito.

[85] Fischhoff B, Broomell SB (2020). Judgment and decision making. Annu. Rev. Psychol..

[86] Anelli F, Nicoletti R, Bolzani R, Borghi AM (2013). Keep away from danger: Dangerous objects in dynamic and static situations. Front. Hum. Neurosci..

[87] Madan CR, Bayer J, Gamer M, Lonsdorf TB, Sommer T (2018). Visual complexity and affect: Ratings reflect more than meets the eye. Front. Psychol..

[88] Nguyen K, McDaniel MA (2015). The picture complexity effect: Another list composition paradox. J. Exp. Psychol. Learn. Mem. Cogn..

[89] Rosenholtz R, Li Y, Nakano L (2007). Measuring visual clutter. J. Vis..

