# Author Correction: Improper weapons are a neglected category of harmful objects

**DOI:** 10.1038/s41598-022-26512-4

**Published:** 2022-12-19

**Authors:** Paolo Frugarello, Elena Rusconi, Remo Job

**Affiliations:** 1 Department of Psychology and Cognitive Science, University of Trento, Corso Bettini 81, 38068 Rovereto, Trento, Italy; 2 Centre for Security and Crime Sciences, University of Trento-University of Verona, Trento, Italy

Correction to: *Scientific Reports* 10.1038/s41598-022-24613-8, published online 22 November 2022

The original version of this Article contained a spelling error in the names of the authors Paolo Frugarello, Elena Rusconi, and Remo Job, which were incorrectly given as Frugarello Paolo, Rusconi Elena, and Job Remo.

The original Article has been corrected.

