# Supplementary material for: Improper weapons are a neglected category of harmful objects
Source: Sci Rep. 2022 Nov 22;12:20078. doi: 10.1038/s41598-022-24613-8 (PMC9684542; doi:10.1038/s41598-022-24613-8)
Supplement: Supplementary file 1 — Supplementary Information. [file 41598_2022_24613_MOESM1_ESM.docx]

**Supplementary table S1.** In the table below, the frequency of use and values for the three visual complexity indices of the various items are listed for the three categories. The following labels indicate the various frequency of use: BA is for "basic frequency," HU stands for "high use," HA stands for "high availability," CO stands for "common," and TS stands for “technical-specialized."

| **Category** | **Frequency of use** | **Feature congestion** | **Subband Entropy** | **Edge density** |
| --- | --- | --- | --- | --- |
| **Proper**  **weapons** |  | | | |
| Mace | **CO** | 2.302 | 1.595 | 0.006581024 |
| Flare gun | **CO** | 1.463 | 1.762 | 0.005973187 |
| Bomb | **HU** | 1.560 | 0.656 | 0.003965638 |
| Dagger | **HA** | 2.110 | 1.433 | 0.00813755 |
| Puncher | **TS** | 3.253 | 2.482 | 0.01251335 |
| Taser | **TS** | 1.859 | 1.711 | 0.004361979 |
| Dynamite | **CO** | 1.800 | 1.143 | 0.003284772 |
| Bullets | **CO** | 4.324 | 3.556 | 0.002559655 |
| Baton | **CO** | 1.596 | 1.253 | 0.004036325 |
| Detonator | **CO** | 2.202 | 1.57 | 0.007300876 |
| Gun | **BA** | 1.468 | 1.266 | 0.001814779 |
| Sword | **HU** | 2.268 | 1.55 | 0.008822751 |
| Swiss knife | **CO** | 2.525 | 2.416 | 0.008417222 |
| Rifle | **HU** | 1.649 | 1.349 | 0.008007948 |
| Assault rifle | **TS** | 1.706 | 1.617 | 0.01644433 |
| **Improper weapons** | **Frequency of use** | **Feature congestion** | **Subband Entropy** | **Edge density** |
| Baseball bat | **TS** | 1.733 | 1.989 | 0.008800217 |
| Cooking thermometer | **TS** | 4.182 | 2.733 | 0.0122516 |
| Cutter | **TS** | 3.278 | 1.980 | 0.005182292 |
| Screwdriver | **HA** | 1.459 | 0.84 | 0.004813547 |
| Hammer | **HU** | 1.793 | 1.828 | 0.007836538 |
| Tube | **HU** | 2.296 | 1.494 | 0.01252841 |
| Firecrackers | **HA** | 2.092 | 1.676 | 0.006449343 |
| Pliers | **HA** | 2.513 | 2.363 | 0.008446828 |
| Chisel | **HA** | 2.074 | 1.540 | 0.003608374 |
| Razor | **HA** | 3.227 | 2.582 | 0.01197727 |
| Scissors | **HA** | 1.488 | 1.215 | 0.0083226 |
| Knife | **BA** | 1.568 | 0.83 | 0.001256142 |
| Blowpipe | **CO** | 1.920 | 1.650 | 0.02172814 |
| Wrench | **TS** | 2.581 | 1.848 | 0.01520024 |
| Saw | **BA** | 2.058 | 1.528 | 0.00531181 |
| **Everyday**  **objects** | **Frequency of use** | **Feature congestion** | **Subband Entropy** | **Edge density** |
| Glass | **BA** | 2.063 | 1.961 | 0.005794885 |
| Hat | **HU** | 2.937 | 2.434 | 0.00068 |
| Jacket | **BA** | 2.266 | 3.359 | 0.006439732 |
| Ball | **HU** | 3.378 | 1.026 | 0.002554088 |
| Bottle | **BA** | 2.318 | 1.188 | 0.005037524 |
| Sock | **HA** | 2.480 | 2.699 | 0.003948521 |
| Shirt | **HA** | 4.125 | 2.221 | 0.001741774 |
| Book | **BA** | 2.678 | 2.606 | 0.01090503 |
| Cap | **HA** | 2.577 | 2.419 | 0.003941517 |
| Undershirt | **HA** | 2.411 | 1.762 | 0.00728678 |
| Bag | **BA** | 2.693 | 1.984 | 0.0152 |
| Skirt | **HU** | 3.631 | 3.637 | 0.01379915 |
| Heel shoe | **HU** | 1.335 | 1.379 | 0.007003649 |
| Jeans | **HU** | 3.285 | 3.484 | 0.01061391 |
| Sneakers | **CO** | 4.483 | 2.094 | 0.02550394 |
